# Supplementary material for: Optical imaging of metabolic dynamics in animals
Source: Nat Commun. 2018 Aug 6;9:2995. doi: 10.1038/s41467-018-05401-3 (PMC6079036; doi:10.1038/s41467-018-05401-3)
Supplement: Supplementary file 1 — Supplementary Information [file 41467_2018_5401_MOESM1_ESM.pdf]

Supplementary Information

"Optical Imaging of Metabolic Dynamics in Animals" by Shi *et al*.

Table of Contents

Supplementary Note 1. Spectral unmixing of D-labeled macromolecules

Supplementary Figure 1. Toxicity of D_2O on cells and *C. elegans*.

Supplementary Figure 2. The development of a three-component unmixing algorithm.

Supplementary Figure 3. Imaging lipogenesis in *C. elegans*.

Supplementary Figure 4. Imaging *de novo* biosynthesis in various mouse organs.

Supplementary Note 1. Spectral unmixing of D-labeled macromolecules

We used chemically extracted, D-labeled total lipids, proteins, and DNA as pure standards to calculate a set of unmixing coefficients (Supplementary Figure 2A and Methods). Applying the three-component unmixing algorithm to dividing cells, we successfully separated the three types of D-labeled macromolecules. In particular, unmixing removed lipid and protein bleed-through in the CD_{DNA} channel and revealed clean D-labeled DNA signal that was only localized in the condensed chromosomes during mitosis (Figure 3A). Signal for D-labeled DNA was very weak in non-dividing cells due to the lack of DNA synthesis and the lower DNA density in the nucleus compared to mitotic cells. Given this fact and that *in vivo* DNA labeling can be achieved by other methods like BrdU staining, we did not focus on imaging the dynamics of DNA metabolism in this study. Instead, we focused on generating signals for D-labeled lipids and proteins, which are much more difficult to optically image *in vivo* using other methods.

To unmix CD_L and CD_P signals, we simplified the three-component unmixing algorithm to a two-component equation and applied it to images acquired at CD_L and CD_P channels (See Methods for details). We validated the effectiveness of unmixing by showing that it abolished the residual bleed-through protein signal in the CD_L images of cells treated with TVB-3155 and the residual bleed-through lipid signal in the CD_P images of cells treated with anisomycin (Figure 3B-D). At the tissue level, we generated more accurate unmixing coefficients using the spectra of pure *in situ* D-labeled lipid and protein (lipid-free) signals obtained by methanol wash (Supplementary Figure 2B and C). We noticed some variation in the level of protein bleed-through into CD_L channel across different tissue types (Supplementary Figure 2C) and adjusted the coefficients accordingly (see Methods for details). Applying the proper calculation to tissue images, we completely removed the protein bleed-through (lipid signal after methanol wash) in the CD_L channel and revealed the genuine distribution of D-labeled lipids and proteins (Supplementary Figure 2D-F). For example, the nucleus became devoid of CD_L signal after unmixing in both cultured cells and tissues (Figure 3E and F). In lipid-rich tissues, unmixing effectively removed the strong lipid-to-CD_P bleed-through and revealed the true D-labeled protein signal (Figure 3G).

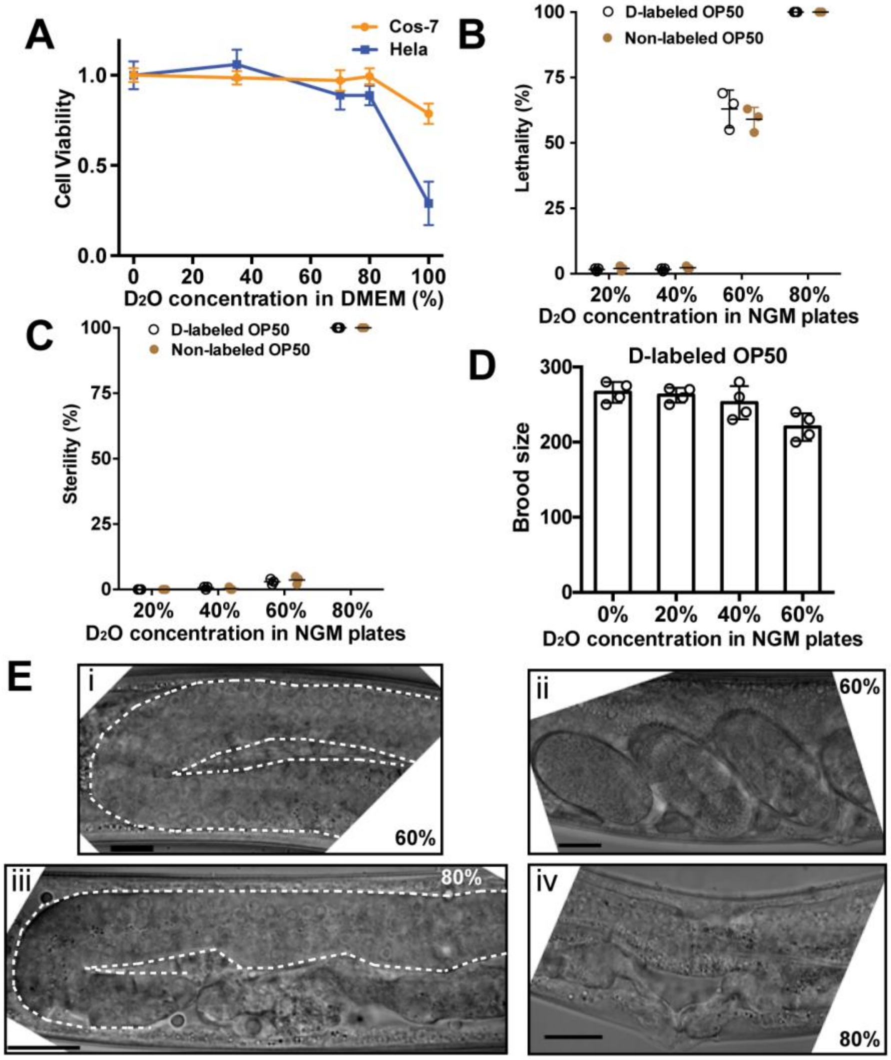

Supplementary Figure 1. Toxicity of D_2O on cells and *C. elegans*. (A) CellTiter-Glo Luminescent Cell Viability Assay, which quantifies ATP production, was performed on Cos-7 and HeLa cells grown in DMEM made of different concentration of D_2O for 48 hours. Three replicates were performed. Mean and s.d. were shown. (B) Lethality was determined by placing hypochlorite-prepared eggs onto NGM plates made of different concentration of D_2O and calculating the percentage of eggs that hatched into normal, moving larva. D-labeled OP50 refers to overnight grown culture of *E. coli* OP50 that were seeded onto the D_2O-containing NGM plates 24 hours before the experiment; non-labeled OP50 refers to the experimental setup, in which bacteria were killed by UV immediately after being placed onto the D_2O plates and thus was not labeled by deuterium. Each group has 50 worms, and three replicates were made. Mean and s.d. were shown. (C) Sterility was determined as the percentage of fourth stage larva (L4) that became sterile after being transferred to D_2O plates. (D) Brood size, which reflects the effects on meiosis, was determined by counting the total number of viable progeny one animal produced. 50 worms were used for each treatment, and four replicates were made. Mean and s.d. were shown. (E) At 60% D_2O concentration, the gonad developed normally (i) and normal embryos were formed (ii) in animals that developed from L4 to adults on D_2O plates. With 80% D_2O, gonad development was disrupted and proliferation of the germ cells appeared to be affected (iii) and no embryos were formed in the uterus (iv). Scale bar = 20 μm.

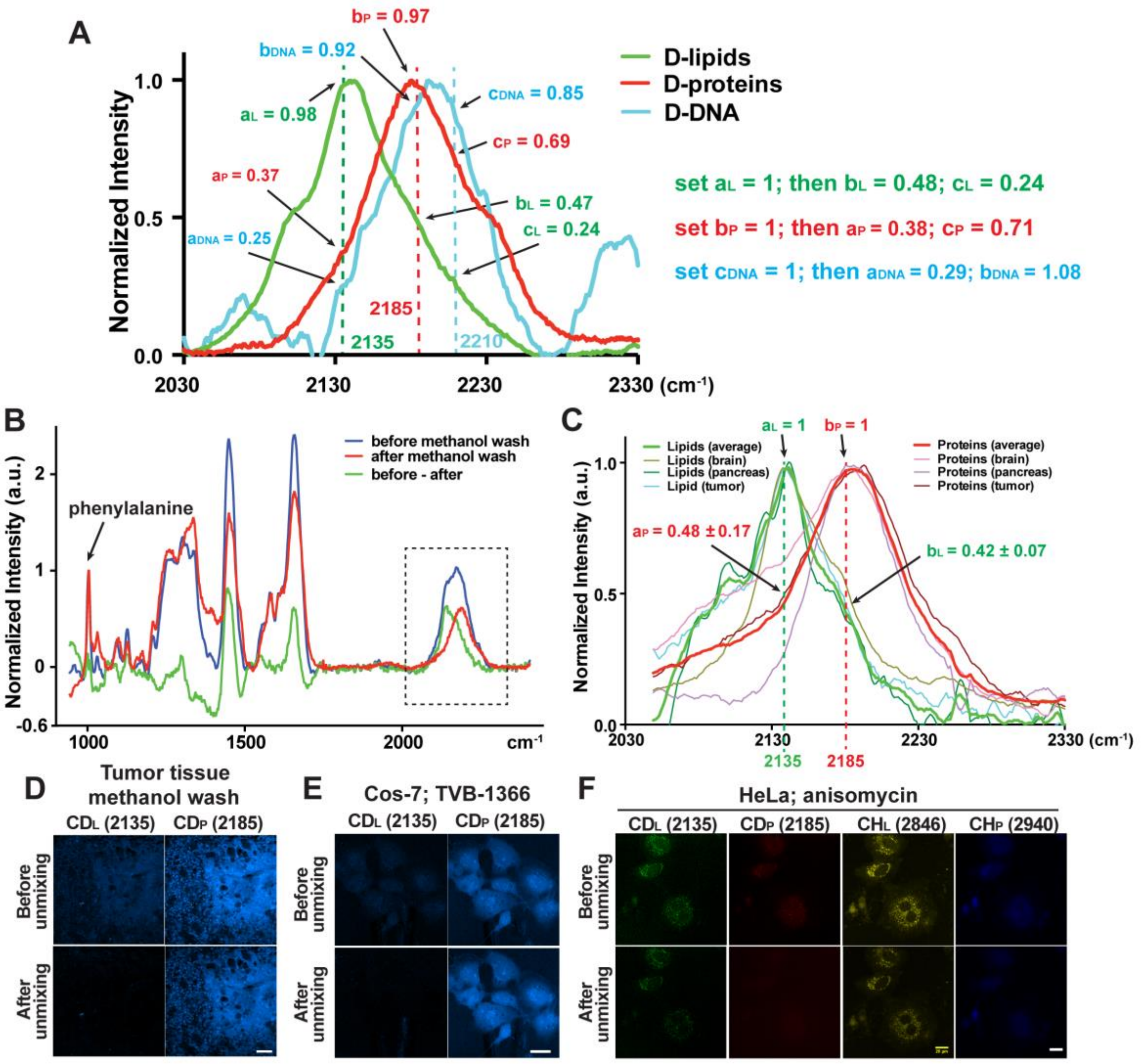

Supplementary Figure 2. The development of a three-component unmixing algorithm. (A) Spontaneous Raman signal of extracted lipids, proteins, and DNA from D_2O-treated HeLa cells. Signals for each type of macromolecules were normalized to their own peaks. Unmixing coefficients were first measured from the spectra and then adjusted by setting a_L, b_P, and c_{DNA} to 1 and scaling the other parameters accordingly. (B) Colon tumor tissues (from mice that drank 25% D_2O for 15 days) before and after methanol wash. Pure deuterium-labeled protein signals (red) were assumed as the methanol-resistant signal, and pure deuterium-labeled lipid signals (green) were calculated as the signal difference before and after methanol wash. All signals were normalized to the phenylalanine peak. (C) Similar methanol wash and signal normalization methods were applied to mouse brain and pancreas tissues, in addition to tumor tissues. The average, normalized pure protein and lipid signal intensities from the three tissues were calculated; mean ± SD for the coefficients were shown. (D) SRS images of methanol-washed colon tumor tissues (taken from tumor-bearing mice that drank 25% D_2O for 15 days) before and after unmixing (a_L = 1; a_P = 0.40; b_P = 1; b_L = 0.51). (E-F) Unmixing was applied to Cos-7 cells grown in 70% D_2O DMEM containing 10 nM TVB-3166 for 24 hours (E) and HeLa cells grown in 70% D_2O DMEM containing 1 μM anisomycin for 24 hours (F). Unmixing equations can be found in the Methods. Scale bar = 20 μm.

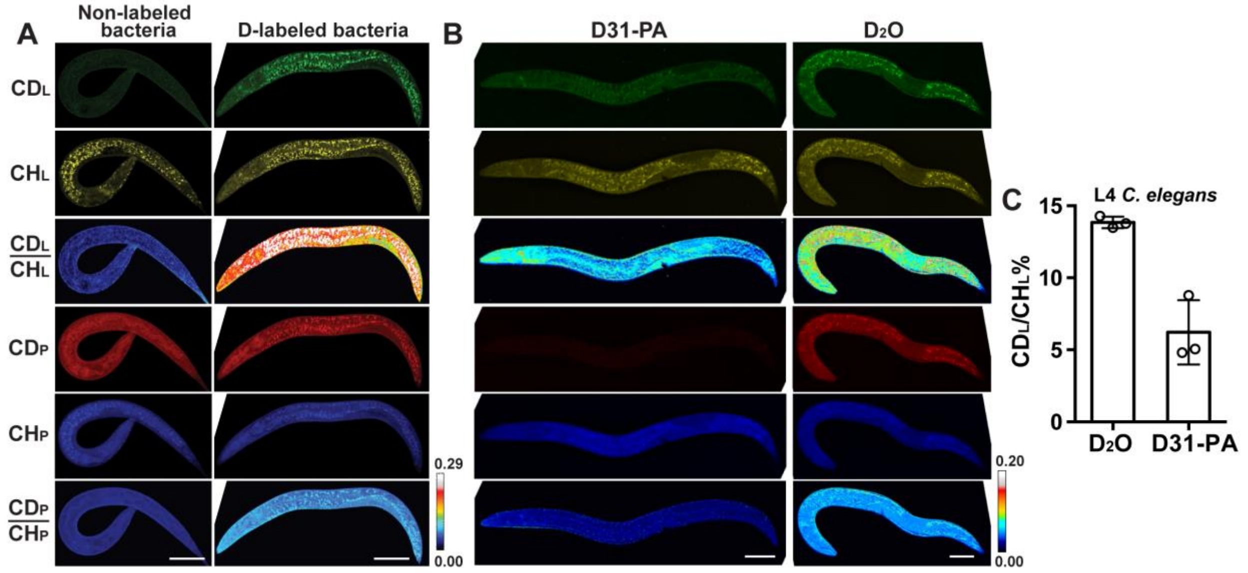

Supplementary Figure 3. Imaging lipogenesis in *C. elegans*. (A) Hypochlorite-prepared eggs of *C. elegans* were placed onto 20% D_2O NGM plates pre-seeded with *E. coli* OP50 and third stage larva grown from those eggs were imaged. For the live bacteria group, OP50 grew on the D_2O plates for 24 hours at room temperature before eggs were placed; for the dead bacteria group, bacteria cells were killed by UV immediately after being seeded onto the D_2O plates, and 24 hours later, eggs were placed onto the plate. Quantification is shown in Figure 4B. (B) OP50 bacterial culture was mixed with 4 mM *D31*-palmitic acid and then seeded onto NGM plates that contains 100% H_2O. *C. elegans* eggs were placed onto those plates and as controls 20% D_2O plates with OP50 seeded the day before. 48 hours later, L4 animals were imaged. The color scale bar represents the scale for CD_L/CH_L and CD_P/CH_P ratios. (C) Comparison of the CD_L/CH_L ratio for animals fed with D31-PA and the ones grown on D_2O plates. Mean ± s.d. are plotted; N = 3 for each treatment. Scale bar = 50 μm.

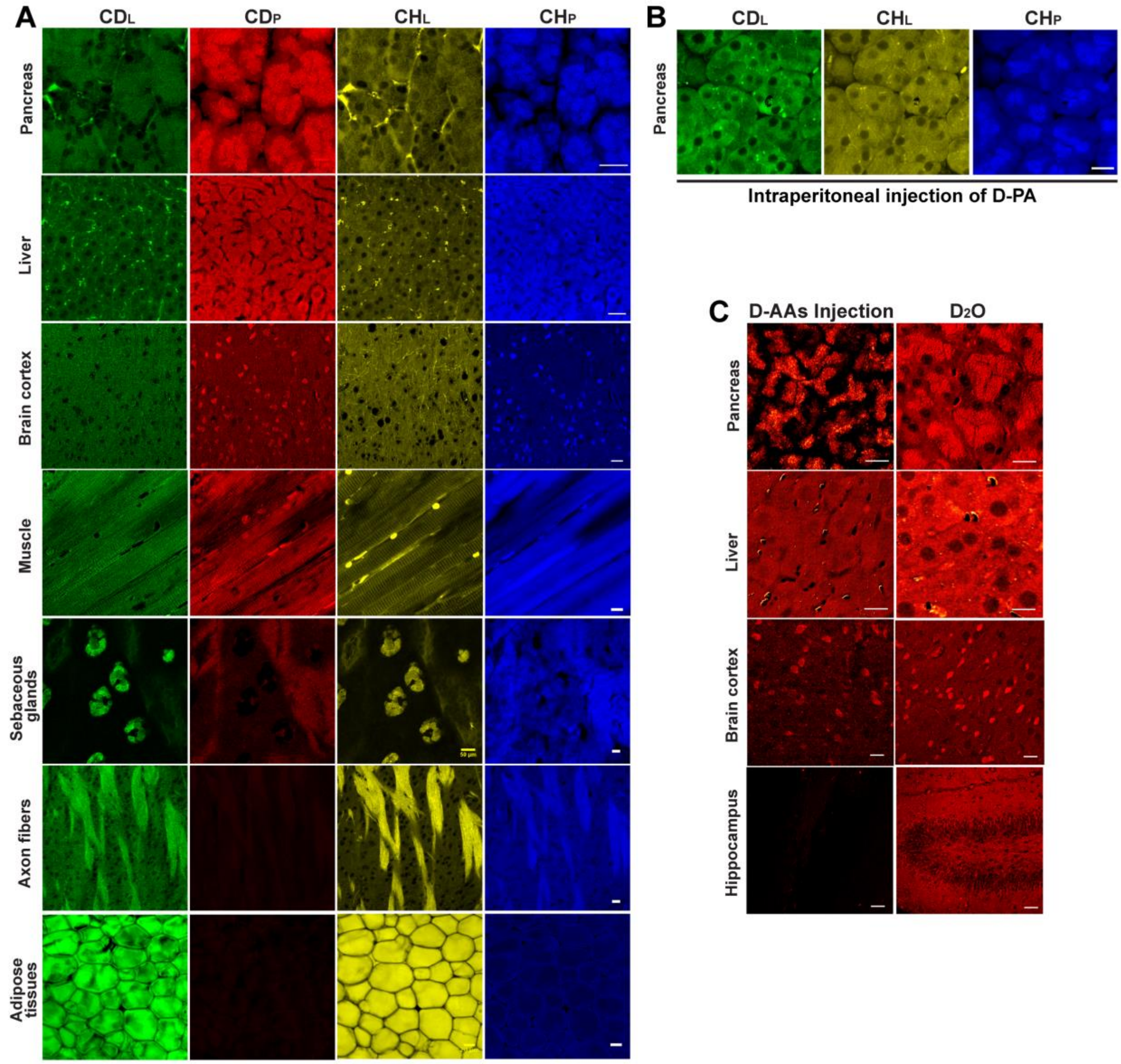

Supplementary Figure 4. Imaging *de novo* biosynthesis in various mouse organs. (A) Various protein-rich tissues, including pancreas, liver, brain cortex, and muscle, were collected from adult mice that drank 25% D_2O for 20 days and then imaged. Lipid-rich sebaceous glands and adipose tissues were harvested from adult mice that drank 25% D_2O for 8 and 15 days, respectively. Myelinated axon fibers of internal capsule were harvested from P11 mouse pups that were fed on milk produced by mother mice drinking 25% D_2O for 6 days before imaging. (B) CD_L signal of the pancreas collected from mice that were intraperitoneally injected with 0.16 ml of 600 mM D-PA emulsion. Tissues were harvested, fixed, and imaged 17 hours after the injection. (C) CD_P signals of various organs from mice that were injected with D-labeled amino acids (d-AAs) *via* carotid artery and mice that drank 25% D_2O for 8 days. Scale bar = 20 μm.
