# Supplementary material for: Optical imaging of metabolic dynamics in animals
Source: Nat Commun. 2018 Aug 6;9:2995. doi: 10.1038/s41467-018-05401-3 (PMC6079036; doi:10.1038/s41467-018-05401-3)
Supplement: Supplementary file 3 — Description of Additional Supplementary Files [file 41467_2018_5401_MOESM3_ESM.pdf]

Description of Additional Supplementary Files

File Name: Supplementary Movie 1
Description: In vivo SRS imaging of D-labeled lipids in the sebaceous glands of a living mouse. SRS recordings at 2135 cm-1 of the sebaceous glands under the ear skin of intact live mice that drank 25% D2O for 9 days.

File Name: Supplementary Movie 2
Description: In vivo SRS imaging of pre-existing lipids in the sebaceous glands of a living mouse. SRS recordings at 2845 cm-1 of the sebaceous glands under the ear skin of intact live mice that drank 25% D2O for 9 days.

File Name: Supplementary Movie 3
Description: Non-resonance background for SRS imaging of D-labeled lipids in the sebaceous glands of a living mouse. SRS recordings at 2135 cm-1 with resonance off, serving as a control for Supplementary Movie 1. C-D signal was no longer generated but the blood flow in the living mouse tissue can be seen because the two-photon absorption of red blood cells is independent of resonance.

File Name: Supplementary Movie 4
Description: SRS live imaging of D-labeled lipids of live C. elegans. SRS recordings at 2135 cm-1 of a live, moving C. elegans larvae (fourth stage) that grew on 20% D2Ocontaining NGM plates for 4 hours.

File Name: Supplementary Movie 5
Description: SRS live imaging of pre-existing lipids of live C. elegans. SRS recordings at 2845 cm-1 of a live, moving C. elegans larvae (fourth stage) that grew on 20% D2Ocontaining NGM plates for 4 hours.

File Name: Supplementary Movie 6
Description: Non-resonance background for SRS live imaging of D-labeled lipids of live C. elegans larvae. SRS recordings at 2135 cm-1 with resonance off, serving as a control for Supplementary Movie 4.
